# Apical-basal distribution of different subtypes of spiral ganglion neurons in the cochlea and the changes during aging

**DOI:** 10.1371/journal.pone.0292676

**Published:** 2023-10-26

**Authors:** Meijian Wang, Shengyin Lin, Ruili Xie

**Affiliations:** 1 Department of Otolaryngology, The Ohio State University, Columbus, OH, United States of Ameirca; 2 Department of Neuroscience, The Ohio State University, Columbus, OH, United States of Ameirca; University of Michigan, UNITED STATES

## Abstract

Sound information is transmitted from the cochlea to the brain mainly by type I spiral ganglion neurons (SGNs), which consist of different subtypes with distinct physiological properties and selective expression of molecular markers. It remains unclear how these SGN subtypes distribute along the tonotopic axis, and whether the distribution pattern changes during aging that might underlie age-related hearing loss (ARHL). We investigated these questions using immunohistochemistry in three age groups of CBA/CaJ mice of either sex, including 2–5 months (young), 17–19 months (middle-age), and 28–32 months (old). Mouse cochleae were cryo-sectioned and triple-stained using antibodies against Tuj1, calretinin (CR) and calbindin (CB), which are reportedly expressed in all type I, subtype I_a_, and subtype I_b_ SGNs, respectively. Labeled SGNs were classified into four groups based on the expression pattern of stained markers, including CR^+^ (subtype I_a_), CB^+^ (subtype I_b_), CR^+^CB^+^ (dual-labeled I_a_/I_b_), and CR^-^CB^-^ (subtype I_c_) neurons. The distribution of these SGN groups was analyzed in the apex, middle, and base regions of the cochleae. It showed that the prevalence of subtype I_a_, I_b_ and dual-labeled I_a_/I_b_ SGNs are high in the apex and low in the base. In contrast, the distribution pattern is reversed in I_c_ SGNs. Such frequency-dependent distribution is largely maintained during aging except for a preferential reduction of I_c_ SGNs, especially in the base. These findings corroborate the prior study based on RNAscope that SGN subtypes show differential vulnerability during aging. It suggests that sound processing of different frequencies involves distinct combinations of SGN subtypes, and the age-dependent loss of I_c_ SGNs in the base may especially impact high-frequency hearing during ARHL.

## Introduction

Type I spiral ganglion neurons (SGN) make up 95% of all SGNs and are the main cells to transmit sound information from the sensory hair cells to the brain [1, 2]. These neurons show diverse physiological properties and were traditionally classified into different subtypes based on their spontaneous firing rate and threshold to sound [3–6]. Specifically, type I SGNs are composed of high spontaneous rate (HSR) neurons with low sound threshold, medium spontaneous rate (MSR) neurons with medium sound threshold, and low spontaneous rate (LSR) neurons with high sound threshold [3]. LSR SGNs show a much broader dynamic range in encoding sound intensity than HSRs [4, 7], and are believed to be important in detecting transient [8] and high intensity signals in noise [9, 10]. Morphologically, these SGNs differ in dendritic fiber caliber [2, 11], cochlear synaptic location and structure on inner hair cells (IHC) [2, 12], and their central projections and synaptic structure in the cochlear nucleus [13–15]. Particularly, HSR neurons have thick fiber diameter and synapse onto the pillar side of IHCs, while LSR neurons have thin fiber diameter and synapse onto the modiolar side of IHCs [2]. Studies also showed that different subtypes of SGNs are not evenly distributed along the frequency axis [5, 16–18], and are differentially altered under various pathological conditions including aging [18–20]. However, the comprehensive distribution pattern of these SGNs across tonotopy and the age-related changes have not been well-characterized in detail.

Recent studies using single-cell RNA sequencing technique identified various molecular markers that define three subtypes of type I SGNs [18, 21, 22]. Of particular interest, calretinin (CR) is expressed in subtype I_a_ SGNs, calbindin (CB) is expressed in subtype I_b_ SGNs, whereas Pou4f1 is expressed in subtype I_c_ SGNs. Additional studies based on synaptic location [18, 21, 23, 24] and physiological property [25] showed that the molecularly identified subtype I_a_, I_b_ and I_c_ SGNs roughly correspond to traditionally classified HSR, MSR and LSR SGNs. These findings make studies possible to conveniently identify SGN cell types and investigate the comprehensive distribution pattern of different SGN subtypes across tonotopic axis.

In this study, we used immunohistochemistry to identify various SGN subtypes in cryo-sectioned cochlea by simultaneously staining three molecular markers of Tuj1, CR, and CB [18, 21, 26]. Four groups of type I SGNs were classified based on the expression pattern of these markers, including CR^+^ (subtype I_a_), CB^+^ (subtype I_b_), CR^+^CB^+^ (dual-labeled), and CR^-^CB^-^ (subtype I_c_) neurons. We found that all type I SGN subtypes showed gradient distribution across frequencies, with the proportions of subtype I_a_, I_b_, and dual-labeled I_a_/I_b_ SGNs decreased from the apex to the base regions of the cochlea, and the proportion of subtype I_c_ SGNs reciprocally increased. The overall SGN density decreased with age, reflecting age-dependent SGN loss. Furthermore, the cell loss preferentially impacted subtype I_c_ SGNs in the base region. The frequency-dependent distribution of SGN subtypes indicates that subtype I_a_ and I_b_ SGNs may contribute more to the processing of low frequency sound, and subtype I_c_ SGNs likely play more roles in the processing of mid-high frequency sound. During aging, preferential loss of I_c_ SGNs in the middle and base cochlear regions may uniquely contribute to the hearing deficit during ARHL, especially at high frequencies.

## Materials and methods

### Animals

Animal experiments were conducted under the guidelines of the protocol #2018A00000055, approved by the Institutional Animal Care and Use Committee of The Ohio State University.

CBA/CaJ mice were acquired from the Jackson Laboratory and maintained at the animal facility at The Ohio State University. Mice of either sex were used at three age groups, including 2–5 months (young), 17–19 months (middle age), and 28–32 months (old), which represent normal hearing, mild and severe ARHL, as characterized in previous studies [27–29]. Experiments were performed in batches, and every batch included mice from all three age groups. Hearing status of all mice in this study was evaluated as previously described [27, 28] by recording auditory brainstem response (ABR) under anesthesia after an I.P. injection of ketamine (100 mg/kg) and xylazine (10 mg/kg). Mice were subsequently decapitated and quickly dissected to retrieve the cochleae, which were fixed in PBS with 4% paraformaldehyde overnight at 4°C and de-calcified in 0.12 M EDTA in 0.1 M PBS for 2–3 days. Cochleae were then cryo-protected in 30% sucrose in PBS, embedded in Cryo-Gel (Cat. #:475237; Instrumedics Inc.) and sectioned along the modiolus axis using a cryostat slicer (Leica CM3050 S, Leica Biosystems), at the thickness of 20 μm.

### Immunohistochemistry

Due to the poor outcome of the immunostaining using Pou4f1 antibody in combination with multiple other markers, we chose to use the molecular markers of CR, CB and Tuj1 to identify different subtypes of SGNs. Cochlea sections were triple-stained using primary antibodies against Tuj1 (rabbit anti-Tuj1, Cat# ab52623; Abcam), CR (guinea pig anti-CR, Cat# 214104; Synaptic Systems), and CB (chicken anti-CB, Cat# 214006; Synaptic Systems). Corresponding secondary antibodies were used including goat anti-guinea pig—Alexa Fluor 488 (Cat# A-11073; Invitrogen), goat anti-rabbit—Alexa Fluor 594 (Cat# A-11037; Invitrogen), goat anti-chicken—Alexa Fluor 647 (Cat# A-32933; Invitrogen), and goat anti-rabbit—Alexa Fluor 750 (Cat# A-21039; Invitrogen). Slices were mounted using DAPI Fluoromount-G mounting medium (Cat# 0100–20; Southern Biotech) to label cell nucleus and improve cell identification. Stained cochleae were imaged using an Olympus FV3000 confocal microscope (Olympus) with the same parameter settings. Z-stack images were acquired across 9 μm in depth at the step of 1.5 μm.

### Confocal imaging and analysis

Image analysis used Imaris version 9.9 (Oxford Instruments). Confocal images from all mice were pooled together to set the threshold blindly in each of the four channels (DAPI, CR, CB, Tuj1), at a level to include all SGNs with visually identifiable labeling above background noise. SGNs with strong or weak marker expression were all identified as positive for the marker. SGN cell bodies were reconstructed using the “Spot” tool based on the Tuj1 staining, and verified by DAPI labeled cell nuclei. Subtype-specificity of each SGN was determined by the staining patterns of the above markers. Specifically, four subgroups of SGNs were quantified among all Tuj1-labelled type I SGNs in the apex, middle and basal portions of the spiral ganglia, including those that express CR (CR^+^), CB (CB^+^), both CR and CB (CR^+^CB^+^), and none of the two (CR^-^CB^-^). SGN density was calculated as the number of identified SGNs divided by the cross-sectional area of the Rosenthal’s canal. A single representative section from one cochlea per mouse was used, and each reported data point represents an individual animal.

### Statistics

Statistical analysis was performed using Prism, version 6.0h (GraphPad Software Inc.). Both one-way and two-way ANOVA test were used to evaluate the effects of frequency region and age on the distribution of SGN subtypes. Tukey’s multiple comparisons test was used for post hoc analyses. Data are presented as mean ± SD.

## Results

### CBA/CaJ mice develop ARHL during aging

**Fig 1:**
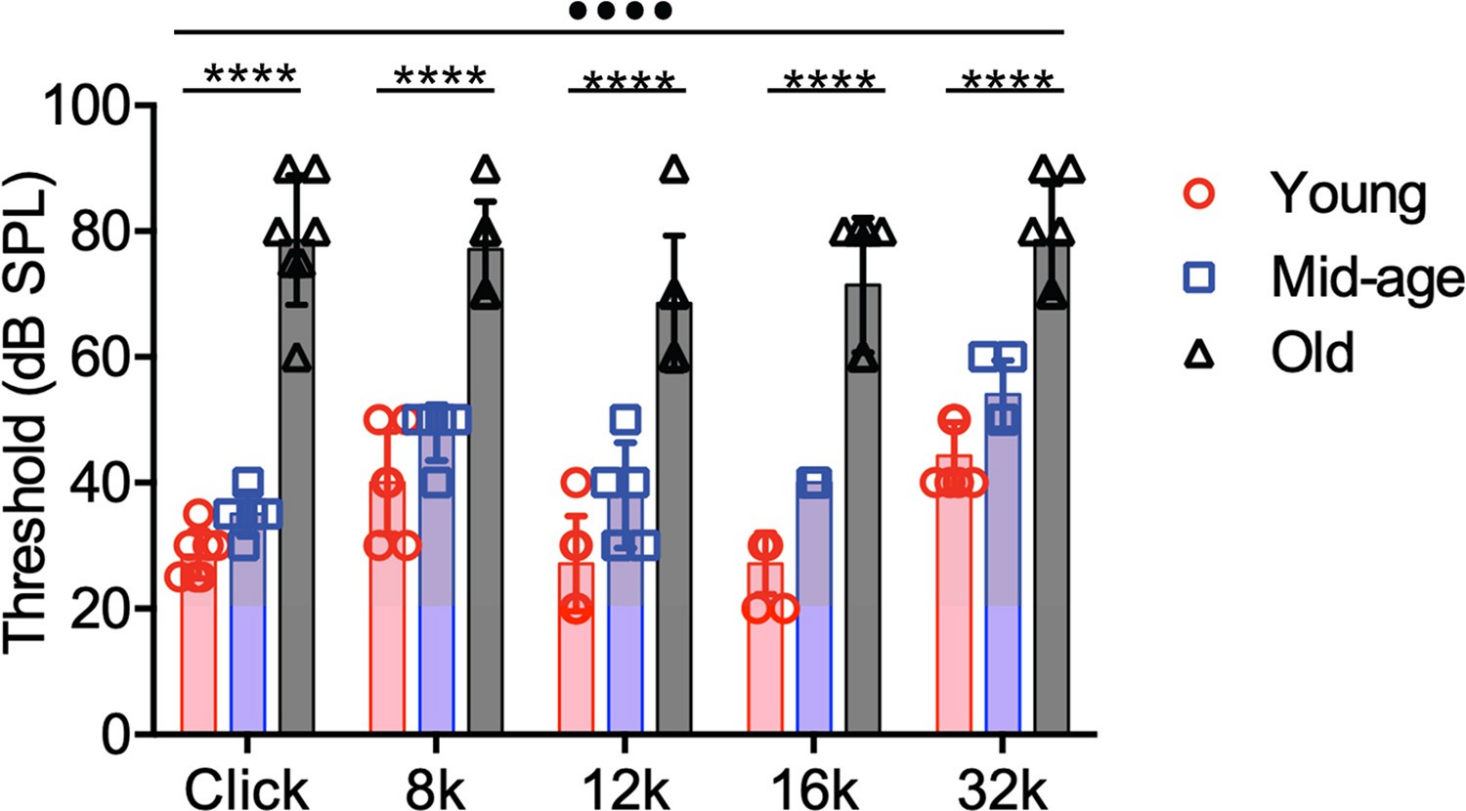
Age-related hearing loss in CBA/CaJ mice. Auditory brainstem response was measured from young (n = 7), middle age (n = 5), and old (n = 7) mice. Two-way ANOVA: age effect: F_(2, 80)_ = 286.8, ••••P < 0.0001; stimulus effect: F_(4, 80)_ = 12.8, P < 0.0001; interaction: F_(8, 80)_ = 1.78, P = 0.0932. Tukey’s multiple comparisons test: P < 0.0001 for all comparisons including young vs. mid-age, mid-age vs. old, and young vs. old groups. Kruskal-Wallis test: ****P < 0.0001.

CBA/CaJ is an inbred mouse train known for its late-onset ARHL during aging [27–29]. We confirmed the hearing status of all mice in this study by measuring their ABRs to clicks and tone bursts at 8, 12, 16, and 32 kHz (S1 Table). As shown in Fig 1, ABR thresholds to all stimuli were slightly (but significantly) evaluated in middle-aged mice (mild hearing loss), and dramatically elevated in old mice (severe hearing loss). The observed changes in hearing of CBA/CaJ mice during aging are consistent with previous reports [27–29].

### Identification of SGN subtypes and their frequency-dependent distribution in young mice

**Fig 2:**
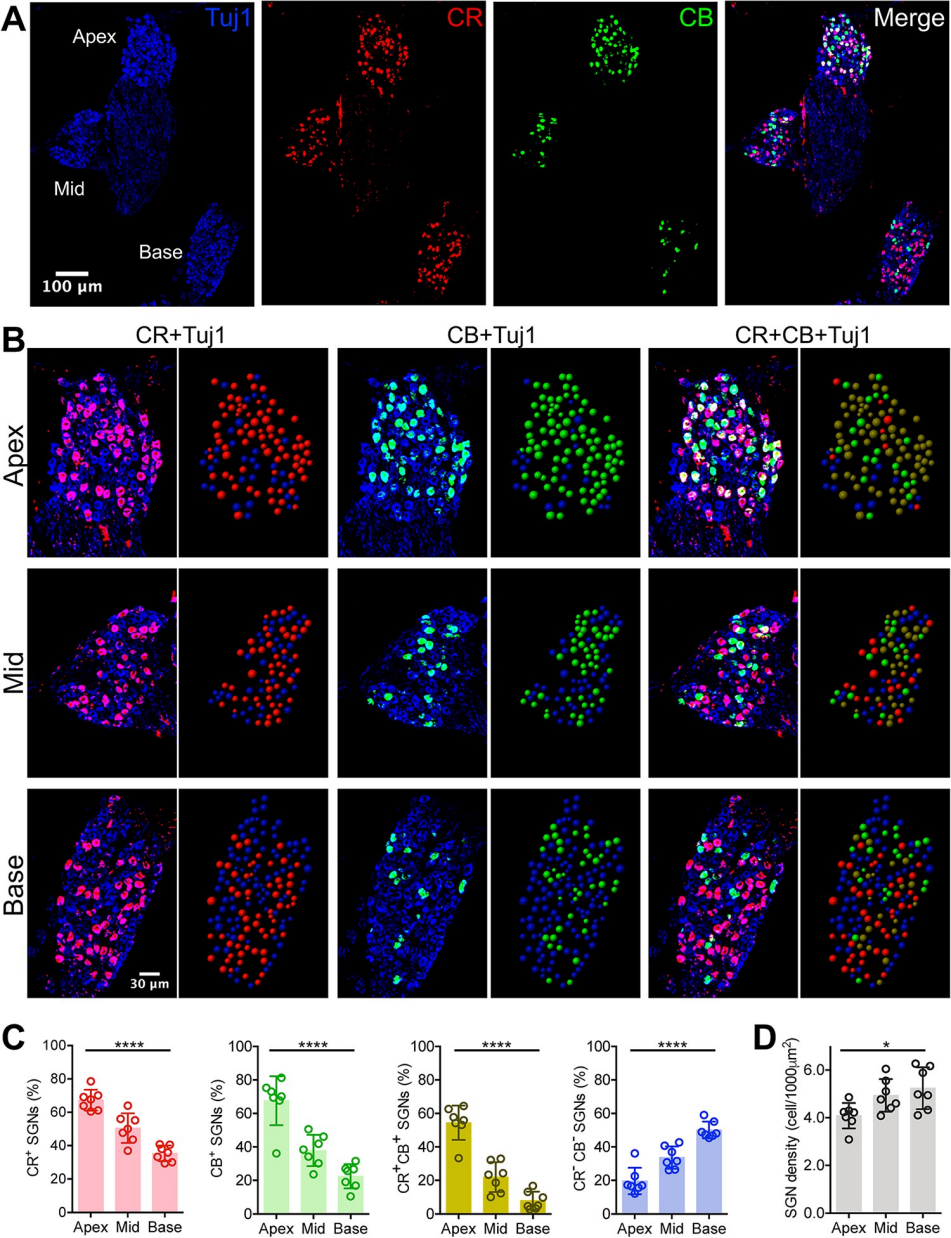
Frequency-dependent distribution of different subtypes of type I SGNs in young mice. (**A**) Cryo-section of an example cochlea (aged p101) stained with Tuj1, calretinin (CR) and calbindin (CB). Notice that CR and CB label different sub-populations of Tuj1^+^ SGNs and their distributions vary across different frequency regions. (**B**) Magnified view of (**A**) to show staining of SGNs in apex, middle and base regions. Color-coded balls on right panels represent identified SGNs. Red: CR^+^ SGNs; green: CB^+^ SGNs. Brown and blue in the rightmost panels represent CR^+^CB^+^ and CR^-^CB^-^ SGNs, respectively. (**C**) Percentage distribution of different subtypes of SGNs in three regions. (**D**) Average SGN cell density in three regions. One-way ANOVA: * P < 0.05; **** P < 0.0001.

We first investigated the distribution of SGN subtypes in young CBA/CaJ mice. As shown in Fig 2A, cell bodies of type I SGNs were clearly labeled by Tuj1-staining [26] in the apex, middle, and base regions of the spiral ganglia in a single cochlea section. Additional CR- and CB-staining labeled different subpopulations of SGNs, as visualized by different colors in the merged panel. To identify and quantify different SGNs, we reconstructed all three regions of the spiral ganglia using balls of different colors to represent the cell bodies of labeled SGNs. As shown in Fig 2B, CR^+^ SGNs are represented as red balls among all Tuj1-labeled neurons in the left two columns (CR+Tuj1 overlap), CB^+^ SGNs are shown as green balls in the middle two columns (CB+Tuj1 overlap). There are relatively more red and green balls in the apex than the middle and base regions, revealing a frequency-dependent distribution of CR^+^ and CB^+^ SGNs. Overlap of all three stained markers (right two columns) showed that many SGNs were double-labeled by CR and CB, which we classified as CR^+^CB^+^ neurons (brown balls). Neurons without CR or CB labeling were classified as CR^-^CB^-^ SGNs, represented as blue balls in the right column, which showed clear increase in prevalence toward the base region.

We summarized the cellular prevalence of different SGNs in 7 young mice (S2 Table), and found that CR^+^, CB^+^ and CR^+^CB^+^ neurons were more abundant in the apex and fewer in the base; while the CR^-^CB^-^ neurons showed an opposite distribution pattern (Fig 2C). On average, the prevalence of CR^+^ neurons was 67 ± 6% in the apex, 51 ± 9% in the middle, and 35 ± 5% in the base region of the spiral ganglia (one-way ANOVA: F_(2, 18)_ = 38.2, P < 0.0001; Tukey’s multiple comparisons test: P < 0.01 for all pairs). The prevalence of CB^+^ neurons was 68 ± 15% in the apex, 38 ± 9% in the middle, and 22 ± 7% in the base region of the spiral ganglia (one-way ANOVA: F_(2, 18)_ = 31.3, P < 0.0001; Tukey’s multiple comparisons test: P < 0.05 for all pairs). Among all CR^+^ and CB^+^ SGNs, there were neurons positive for both markers, and the prevalence of these double-labeled CR^+^CB^+^ neurons was 54 ± 10% in the apex, 22 ± 9% in the middle, and 8 ± 5% in the base region of the spiral ganglia (one-way ANOVA: F_(2, 18)_ = 56.1, P < 0.0001; Tukey’s multiple comparisons test: P < 0.05 for all pairs). In contrast, the prevalence of CR^-^CB^-^ neurons was 20 ± 8% in the apex, 34 ± 7% in the middle, and 50 ± 5% in the base regions of the spiral ganglia (one-way ANOVA: F_(2, 18)_ = 37.0, P < 0.0001; Tukey’s multiple comparisons test: P < 0.01 for all pairs). As a reference, the overall cell density of SGNs was 4.1 ± 0.5 cell/1000 μm^2^ in the apex, 4.9 ± 0.7 cell/1000 μm^2^ in the middle, and 5.2 ± 0.9 cell/1000 μm^2^ in the base regions of the spiral ganglia (Fig 2D; one-way ANOVA: F_(2, 18)_ = 5.0, P = 0.019; Tukey’s multiple comparisons test: P < 0.05 between apex and base, P > 0.05 for other pairs). The results suggest that sound encoding at different frequencies are achieved by different populations of SGN subtypes, in which CR^+^ and CB^+^ SGNs play a larger role in encoding low frequency sound and CR^-^CB^-^ SGNs play a larger role in encoding high frequency sound. Given that different SGN subtypes vary in sound threshold [3] and dynamic range [4, 7], it is expected that sound levels are also processed differently along tonotopy, in which most low frequency SGNs have optimized processing for low intensity sound and a relatively higher percentage of high frequency SGNs favor high intensity sound.

### Distribution of SGN subtypes changes with age

**Fig 3:**
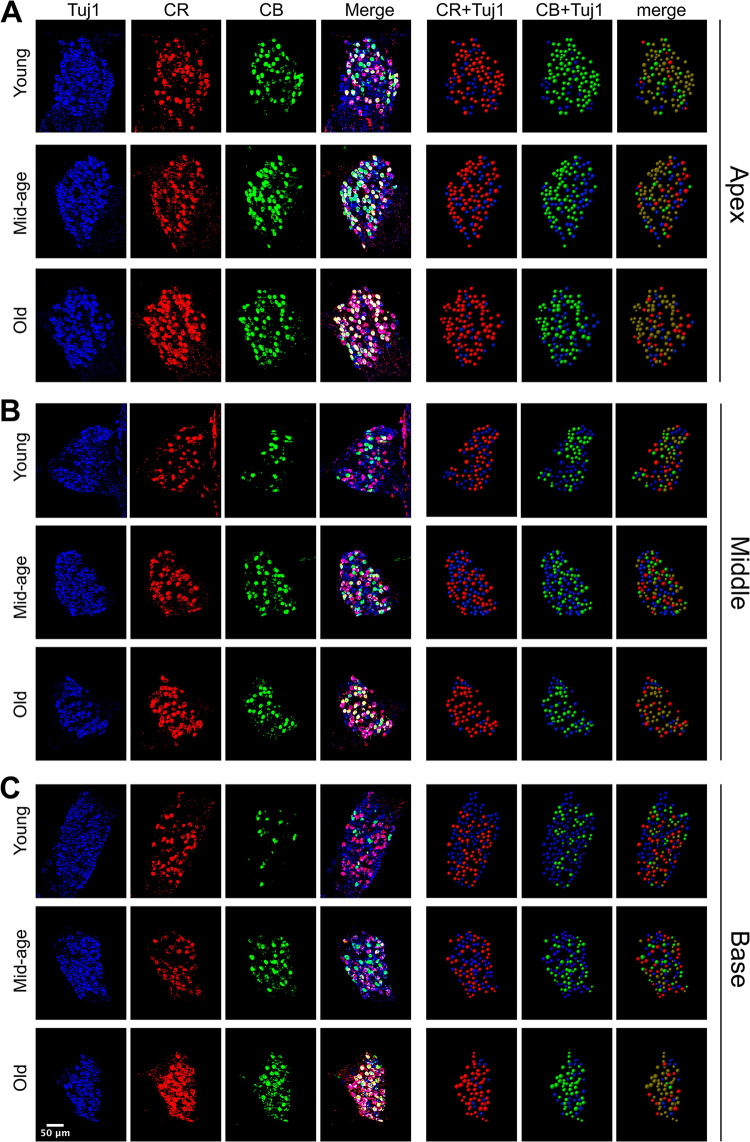
Age-dependent changes in the distribution of different subtypes of type I SGNs. (**A-C**) Staining of example cochleae from young, middle-age, and old mice across three frequency regions in the apex (**A**), middle (**B**), and base (**C**). Color-coded balls on right panels represent identified SGNs. Brown and blue balls in the rightmost column represent CR^+^CB^+^ and CR^-^CB^-^ SGNs, respectively.

To investigate the age-dependent changes of SGN subtypes, we extended our study to include 5 middle-age and 7 old mice (S2 Table). Representative examples of cochlea staining are shown in Fig 3, including the apex (Fig 3A), middle (Fig 3B) and base (Fig 3C) regions of the spiral ganglia from all three age groups. While the prevalence of CR^+^ and CB^+^ neurons remained relatively steady during aging in the apex region (Fig 3A), both were clearly increased with age in the middle (Fig 3B) and base (Fig 3C) regions. Consistently, the prevalence of double labeled CR^+^CB^+^ neurons (brown balls in merged panels) were also increased. In contrast, the prevalence of CR^-^CB^-^ neurons (blue balls in merged panels) were decreased with age in the middle and base regions.

**Fig 4:**
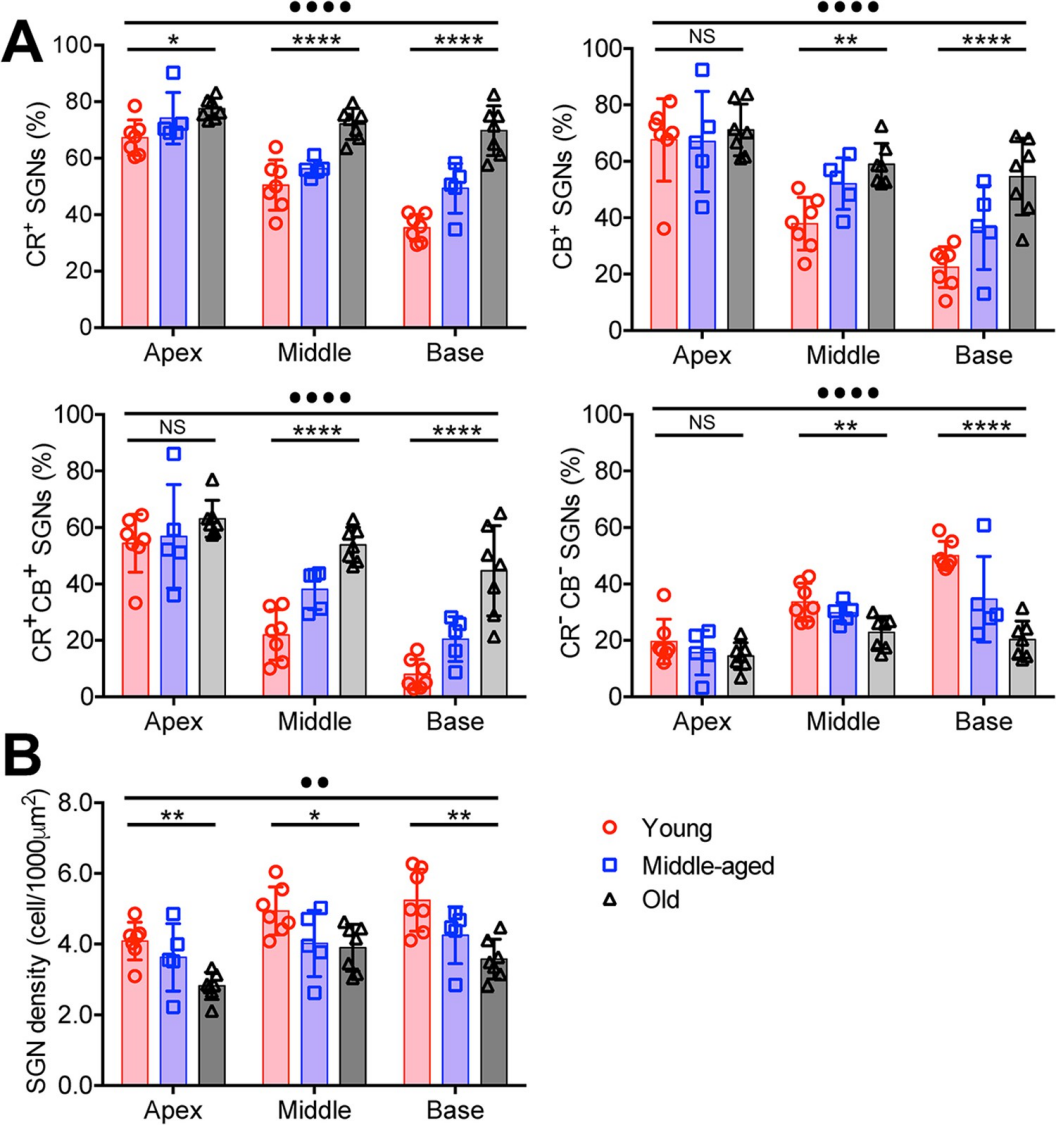
Summary distribution of different subtypes of type I SGNs during aging. (**A**) Summary distributions of CR^+^, CB^+^, CR^+^CB^+^, and CR^-^CB^-^ SGNs during aging in the apex, middle and base regions of the cochleae. (**B**) Changes in the average SGN cell density during aging across three frequency regions. One-way ANOVA: * P < 0.05; ** P < 0.01; **** P < 0.0001; NS: not significant. Two-way ANOVA: •• P < 0.01; ••••P < 0.0001.

Specifically, as shown in Fig 4A, the prevalence of CR^+^ neurons was significantly increased with age in all three regions, including the apex (one-way ANOVA: F_(2, 16)_ = 4.7; P = 0.025), middle (one-way ANOVA: F_(2, 16)_ = 20.0; P < 0.0001), and the base region (one-way ANOVA: F_(2, 16)_ = 36.8; P < 0.0001). Two-way ANOVA showed significant main effects of age and frequency region, and Tukey’s multiple comparisons test showed significant difference cross frequency regions in the young and middle-aged groups (P < 0.001), but not in the old group (P > 0.05). The prevalence of CB^+^ neurons showed no change with age in the apex region (one-way ANOVA: F_(2, 16)_ = 0.17; P = 0.847), but was significantly increased in the middle (one-way ANOVA: F_(2, 16)_ = 10.8; P = 0.0011) and the base region (one-way ANOVA: F_(2, 16)_ = 12.5; P = 0.0005). Two-way ANOVA showed significant main effects of age and frequency region, and Tukey’s multiple comparisons test revealed significant differences among age groups in the middle (P < 0.01) and base (P < 0.0001), but not in the apex region (P > 0.05). Similarly, the prevalence of CR^+^CB^+^ neurons showed no change in the apex region (one-way ANOVA: F_(2, 16)_ = 1.00; P = 0.390), but was significantly increased in the middle (one-way ANOVA: F_(2, 16)_ = 31.2; P < 0.0001) and the base region (one-way ANOVA: F_(2, 16)_ = 19.8; P < 0.0001). Two-way ANOVA showed significant main effects of age and frequency region, and Tukey’s multiple comparisons test showed significant differences among age groups in the middle (P < 0.0001) and base (P < 0.0001), but not in the apex region (P > 0.05). Finally, the prevalence of CR^-^CB^-^ neurons in the apex region was not changed with age (one-way ANOVA: F_(2, 16)_ = 1.04; P = 0.378), but significantly decreased in the middle (one-way ANOVA: F_(2, 16)_ = 6.43; P = 0.0089) and the base region (one-way ANOVA: F_(2, 16)_ = 18.6; P < 0.0001). Two-way ANOVA showed significant main effects of age and frequency region, and Tukey’s multiple comparisons test showed significant differences among age groups in the middle (P < 0.05) and base (P < 0.0001), but not in the apex region (P > 0.05).

Overall, SGN density (S3 Table) was significantly decreased with age, reflecting the loss of SGNs during aging (Fig 4B). In the apex region, the average SGN density was 4.1 ± 0.5 in young, 3.6 ± 1.0 in middle-age, and 2.8 ± 0.4 cell/1000 μm^2^ in old mice (one-way ANOVA: F_(2, 16)_ = 7.4; P = 0.0054); In the middle region, the average SGN density was 4.9 ± 0.7 in young, 4.0 ± 0.9 in middle-age, and 3.9 ± 0.7 cell/1000 μm^2^ in old mice (one-way ANOVA: F_(2, 16)_ = 3.9; P = 0.040); In the base region, the average SGN density was 5.2 ± 0.9 in young, 4.2 ± 0.8 in middle-age, and 3.6 ± 0.6 cell/1000 μm^2^ in old mice (one-way ANOVA: F_(2, 16)_ = 8.6; P = 0.0029). Two-way ANOVA showed significant main effects of age and frequency region (age effect: F_(2, 48)_ = 18.4, P < 0.0001; frequency region effect: F_(2, 48)_ = 8.1, P = 0.0010; interaction: F_(4, 48)_ = 0.65, P = 0.629).

The results showed that during aging, the overall number of SGNs gradually decreased in all three frequency regions due to cell loss (Fig 4B). Furthermore, the age-dependent SGN loss is more prominent in CR^-^CB^-^ neurons, especially in the middle and base regions of the spiral ganglia (Fig 4A). Accordingly, the prevalence of CR^+^ and CB^+^ are relatively increased, leading to altered composition of different SGN subtypes across different frequency regions of the cochlea. Given that different SGN subtypes have distinct physiological function in processing sound, such altered distribution during aging is expected to differentially impact hearing across different frequencies, especially contributing to the typical high frequency hearing loss in ARHL.

## Discussion

Using immunohistochemistry with three molecular markers, this study characterized the distribution patterns of different subtypes of type I SGNs and their age-related changes in the cochlea across three frequency regions (apex, middle and base). The observed gradient distribution of various subtypes along frequency axis suggest that these SGNs play different but complementary roles in processing various sound frequencies. Particularly, subtype I_a_ and I_b_ SGNs likely dominate in the low frequencies, whereas subtype I_c_ SGNs reciprocally contribute more in high frequencies. Such frequency-dependent distribution of SGN subtypes was observed in cats [16] and gerbils [5, 20] by sampling auditory nerve fibers using single unit recording, all of which reported higher percentage of HSR fibers in low frequencies and higher percentage of LSR fibers in high frequencies. However, two studies in CBA/CaJ mice did not find any apparent relationship between fiber spontaneous rate and characteristic frequency [30, 31]. As single unit recording technique relies on spike activity to detect and classify auditory nerve fibers, sampling bias may occur due to the lower probability in detecting less active fibers and less chance of accessing thinner fibers, which would affect the overall representation of the fiber population in these experiments. Conversely, studies analyzing the fiber diameter of the cochlear nerve in mice [17] and monkeys [32] showed that fibers from the base turn have thinner diameter on average than those from the apical turn. Since LSR SGNs have thinner fiber diameter than HSR SGNs [2, 11], these studies support a relatively higher percentage of LSR SGNs in the high frequency region. Consistently, the gradient distribution pattern of different SGNs was also observed at the gene expression level from single-cell RNA sequencing of SGNs [18].

The observed loss of subtype I_c_ SGNs in middle-age and old mice agrees with the idea that LSR neurons are more vulnerable [19] and are preferentially damaged during aging [27, 29, 33]. These SGNs have high threshold and are critical in the processing of high intensity signals, especially in noisy environment. Selective loss of these neurons, or even just the loss of their peripheral synapses with intact cell bodies (termed cochlear synaptopathy), is believed to underlie the phenomena of hidden hearing loss [34, 35], under which the hearing threshold remains normal in quiet condition but signal detection in noisy environment is compromised. Remarkably, we found that the preferential loss of I_c_ SGNs during aging is progressively more profound in the base region of the cochlea (Fig 4A). ARHL usually starts from the high frequency region and progresses toward the low frequency region [36, 37], which occurs in parallel with the gradient loss of I_c_ SGNs along the frequency axis over time (Fig 4A). During the process, cochlear synaptopathy is known to occur long before any detected threshold changes and SGN loss [29]. It suggests that pathological changes of type I_c_ cochlear synapses and SGNs in the base region may uniquely drive the high frequency hearing loss during aging.

Despite the widely observed vulnerability in LSR SGNs under pathological conditions [19, 33, 38, 39], the underlying mechanisms of their preferential damage remains unclear. Given that IHCs are each innervated by all subtypes of SGNs, it is unlikely that IHC loss or reduced amplification due to outer hair cell loss during ARHL [29, 40] would contribute to the differential damage of specific SGN subtypes. One possible explanation may lie in their distinct molecular identity of not expressing CR and CB, two major calcium binding proteins in regulating intracellular calcium [41, 42]. Calcium is a ubiquitous intracellular messenger that controls crucial cellular function including life and death. The concentration of calcium is tightly regulated through various pathways, with high calcium being toxic under pathological conditions [43, 44]. CR and CB are among the most abundant calcium binding proteins in the brain that chelate free calcium and maintain intracellular calcium at a low level, which is protective. Lacking CR and CB may impose additional risk to subtype I_c_ SGNs under challenging conditions, leading to the preferential damage of synapses [27] and eventually cell death [19, 33, 38, 39].

One limitation of this study was that the classification of SGN subtypes was based on the expression patterns of only three molecular markers, including Tuj1, CR and CB. These markers have been recognized to label all type I, subtype I_a_ and subtype I_b_ SGNs [18, 21, 26], respectively. Due to the technical difficulty we had with the Pou4f1 antibody in the staining, we used the Tuj1 antibody instead to identify all type I SGNs, and classified the cells without CR and CB expression as subtype I_c_ SGNs. These CR^-^CB^-^ neurons are expected to express Pou4f1, but may not represent all Pou4f1-expressing SGNs due to the fact that some neurons express more than one of these markers [18, 21]. Indeed, we found many SGNs that express both CR and CB and analyzed these neurons in the dual-labeled I_a_/I_b_ group. Nonetheless, SGNs are far more heterogenous in their molecular identity beyond these selected markers [18, 21, 22], and should not be considered as a simple collection of exclusively distinct subpopulations of neurons. The classification of four type I SGN subpopulations in this study is by no means an ideal way to unambiguously identify functionally distinct type I SGN subtypes, but rather a simplified approach to provide a convenient and valid method to systematically characterize the general distribution patterns of different SGNs and their age-related changes. Finally, about 5% of the SGN population are type II neurons, which play significant roles in auditory nociception [45, 46]. These SGNs were not investigated in this study, and it remains unclear if they are differentially distributed along tonotopy and how their distribution change during ARHL.

## Supporting information

S1 Table Hearing threshold of all mice assessed by aditory brainstem response (ABR), using click and tones of different frequencies. Each row represents a single mouse. All mice in three age groups are listed, including young, mid (middle aged) and old mice.
(XLSX)

S2 Table Percentage of four subpopulations of type I SGNs as classified in the study from all mice. Each subpopulation includes three age groups of mice at young (Y), middle aged (M), and old (O) ages.
(XLSX)

S3 Table SGN cell density from three cochlea regions. Three age groups of mice are including: young (Y), middle aged (M), and old (O) mice.
(XLSX)
